# The Mandate for a Global “One Health” Approach to Antimicrobial Resistance Surveillance

**DOI:** 10.4269/ajtmh.18-0973

**Published:** 2019-01-02

**Authors:** Siddhartha Thakur, Gregory C. Gray

**Affiliations:** 1 College of Veterinary Medicine, NC State, Raleigh, North Carolina;; 2 Division of Infectious Diseases, Duke Medicine, Global Health Institute, and Nicholas School of the Environment, Duke University, Durham, North Carolina

In 1940, one year before the first administration of penicillin in man, two members of the team who discovered the drug revealed that resistance to penicillin already existed.^1^ Since then, as antimicrobial resistance (AMR) has progressed in the wake of exponential antimicrobial use, scientists have raced against extraordinarily efficient microbial gene dissemination and evolution to provide effective antimicrobial therapeutics. Today, with the existence of genes resistant to every natural and synthetic antimicrobial compound, national surveillance systems track AMR in human and animal populations to deepen our understanding of resistance and find ways to circumvent it. Although we have established surveillance systems across North America and Europe, pathogens do not respect international boundaries, and the emergence of resistance in any country poses a worldwide threat. In this issue of the *AJTMH*, Hedman et al.^2^ report spillover of AMR to developing world settings with no prior history of agricultural antimicrobial use. We are reminded that surveillance must become a global “One Health” effort if we are to solve one of today’s most significant threats to human, animal, and environmental health.

Antimicrobial resistance has reached its tipping point, and some are saying we are now in the post-antibiotic era. Recent reports have highlighted this trend, including the emergence of multiple plasmid-mediated colistin resistance genes in human and animal pathogens,^3^ spread of metallo-beta-lactamase-1 in India,^4^ and the emergence of plasmid-mediated carbapenem-resistant *Enterobacteriaceae* in swine for the first time in the United States.^5^ Leading world health agencies consider the threat of AMR as paramount and recognize its complex causation: expanding human and domestic animal populations; increased globalization, international trade, and demand for animal source foods; and all-too-easy access to antimicrobials in both developed and developing countries. The proficiency of genome evolution via horizontal gene transfer and the emergence of new forms of resistance have compounded the lack of new antibiotic discovery and development, while intensifying the threat posed by drug-resistant pathogens. By 2050, an estimated 10 million human lives per year will be at risk if we fail to attenuate the rise of drug resistance, and critical medical procedures such as administration of cancer chemotherapy, joint replacement, and gastrointestinal surgery may be associated with increasing morbidity.^6^ The increase in AMR burden correlates with a 65% increase in antimicrobial consumption in humans between 2000 and 2015 in 76 countries^7^ and administration of 63,000 tons in animals in 2010, with a projected 67% increase in consumption by 2030.^8^

Antimicrobial resistance poses a particularly significant threat to low- and middle-income countries. This is due not only to the health-care challenges these countries face, but also to an increase in small-scale intensive animal production, exacerbated by poor sanitation infrastructure. The findings reported by Hedman et al. in this issue exemplify this problem and the difficulty of understanding the complicated dynamics of AMR transmission between humans and animals sharing the same environment. The researchers investigated the prevalence of CTX-M extended-spectrum beta-lactamases in chickens from small-scale poultry farms and in children living on the farms in rural Ecuador. CTX-M-mediated cephalosporin resistance was seen in bacteria both in commercially bred “broiler” chickens treated with high levels of antibiotics and in free-grazing animals that had no direct exposure to antibiotics. Resistance was also detected in bacteria from children in the community. After phylogenetic analysis, the authors reported a shared evolutionary history between chicken and human samples. Hedman et al., thus, provide valuable insight into the rise of phenotypic resistance and avian-to-human spillover in areas that have previously reported low AMR levels in both poultry and humans.

Altogether, the data provided by Hedman et al. support a familiar narrative: gene exchange is a property of bacteria that efficiently enables the transmission of resistance between animals and humans. Of particular importance to surveillance systems, the study also highlights the pivotal role of the environment in AMR transmission. The ability of the environment to act as a reservoir for resistance is not a new concept and may have promoted the potential spillover event described by Hedman et al. in Ecuador. Indeed, the environmental AMR resistome consists of more than one million distinct bacterial species, which markedly exceeds the number of species that infect human and animal populations.^9^

Despite the knowledge of environmental influences on AMR, current surveillance systems often neglect environmental sampling. It is now crucial that we re-emphasize the role that the environment plays as a reservoir and in maintaining AMR genes as we establish surveillance systems to combat AMR. We know that many of the resistance mechanisms we see in veterinary clinics and animal production systems likely have environmental origins. Recently, we have reported horizontal dissemination of resistance determinants in multiple *Salmonella* serotypes across commercial swine farms following manure application.^10^ In addition, numerous studies have reported very little difference in the shedding of drug-resistant bacterial strains between animals raised under organic or antimicrobial-free production systems.^11–14^ Combined with studies such as that conducted by Hedman et al., these findings demonstrate the need to apply a One Health approach and study environmental reservoirs more closely, rather than focusing only on the resistance that arises following antimicrobial administration.^15–18^

Importantly, combating AMR will also require global cooperation because decreasing the use of antimicrobials only in one population or in one country will not necessarily attenuate the spread of resistant strains. Collignon et al. conducted a multivariate analysis based on antimicrobial consumption data from 63 countries to determine the role of anthropological and socioeconomic factors in the global spread of AMR.^19^ At the country level, the authors suggested that improving sanitation, ensuring good governance, better access to clean water, increasing expenditure on improving public health care, and better regulation of the private health sector were all required for reducing AMR. Factors including poor sanitation, warmer temperatures, and higher corruption levels were consistently associated with a higher prevalence of AMR strains. Without harmonizing surveillance between nations, we will never know the extent of the AMR challenge, nor will we be able to combat it effectively. We are already taking strides to ameliorate this problem. In 2015, the World Health Organization launched the Global AMR Surveillance System (GLASS) to establish a standardized GLASS. So far, 40 countries are participating,^20^ and by collecting and analyzing AMR, epidemiological, clinical, and population-based data from these countries, systems such as GLASS can generate actionable data, improve analysis, influence policy decisions, and ultimately reduce the burden of AMR worldwide.
